# Emergence of dengue at high altitude: characterization of the 2024 outbreak in Cochabamba, Bolivia

**DOI:** 10.1186/s12985-026-03117-1

**Published:** 2026-03-03

**Authors:** Paola Mariela Saba Villarroel, Selin Sen, Raphaëlle Klitting, Laura Pezzi, Geraldine Piorkowski, Norma Villavicencio Siles, Sineewanlaya Wichit

**Affiliations:** 1 Department of Clinical Microbiology and Applied Technology, Faculty of Medical Technology, Mahidol University, Nakhon Pathom, Thailand; 2 Viral Vector Joint unit and Joint Laboratory, Mahidol University, Nakhon Pathom, Thailand; 3 Unité des Virus Émergents (UVE: Aix-Marseille Univ, Università di Corsica, IRBA), IRD 190, INSERM 1207, Marseille, France; 4 Centre National de Référence des Arbovirus, INSERM-IRBA, Marseille, France; 5 Servicio Departamental de Salud (SEDES), Cochabamba, Bolivia

**Keywords:** Dengue virus, Cochabamba, Bolivia, High altitude, Climate change, DENV-2

## Abstract

Dengue virus (DENV) transmission in Bolivia has historically been restricted to tropical lowland regions, where the primary vector *Ae. aegypti* is endemic. In recent years, however, the vector has expanded into high-altitude areas. In 2024, Cochabamba, located at ~ 2,550 m above sea level, experienced its largest recorded dengue outbreak. We analyzed data from 9,576 suspected dengue cases reported between January and July 2024, of which 5,923 were laboratory-confirmed. A subset of DENV-positive samples underwent whole-genome sequencing and phylogenetic analysis. Dengue cases were detected in municipalities located at elevations up to 2,719 m, with the highest number reported in Cochabamba city (4,104/5,923, 69.3%; 2,558 m) and the highest incidence observed in Capinota (839 cases per 100,000 population; 2,386 m). Molecular analyses indicated predominant circulation of DENV-2 genotype II clade F.1.1.2. Phylogenetic reconstruction identified two distinct clades closely related to viruses previously detected in southern Brazil, suggesting multiple independent introductions into the department in late 2023. However, the limited availability of genomic data from Bolivia and neighboring countries limits precise identification of the outbreak’s origin. This study provides the first characterization of the 2024 dengue outbreak in Cochabamba, an exceptional event of substantial DENV circulation at high altitude, likely facilitated by the expanding geographic range of *Ae. aegypti*, potentially driven by climate change.

**Supplementary Information:**

The online version contains supplementary material available at https://doi.org/10.1186/s12985-026-03117-1.

## Introduction

Dengue virus (DENV), a member of the *Orthoflavivirus* genus within the *Flaviviridae* family, comprises four serotypes (DENV-1 to DENV-4) [1], each further subdivided into several genotypes. Among these, DENV-2 includes six genotypes, with genotype II, also known as Cosmopolitan, being the most widespread and genetically diverse [1, 2].

DENV is endemic in most tropical and subtropical regions of the world, where its primary mosquito vector, *Aedes (Ae.) aegypti*, is established. In the Americas, dengue incidence has risen sharply in recent years. Significantly, 2024 recorded a historic burden, with more than 13 million suspected cases and over 7 million laboratory-confirmed infections. The virus also caused outbreaks in previously unaffected areas in Bolivia [3].

**Fig. 1:**
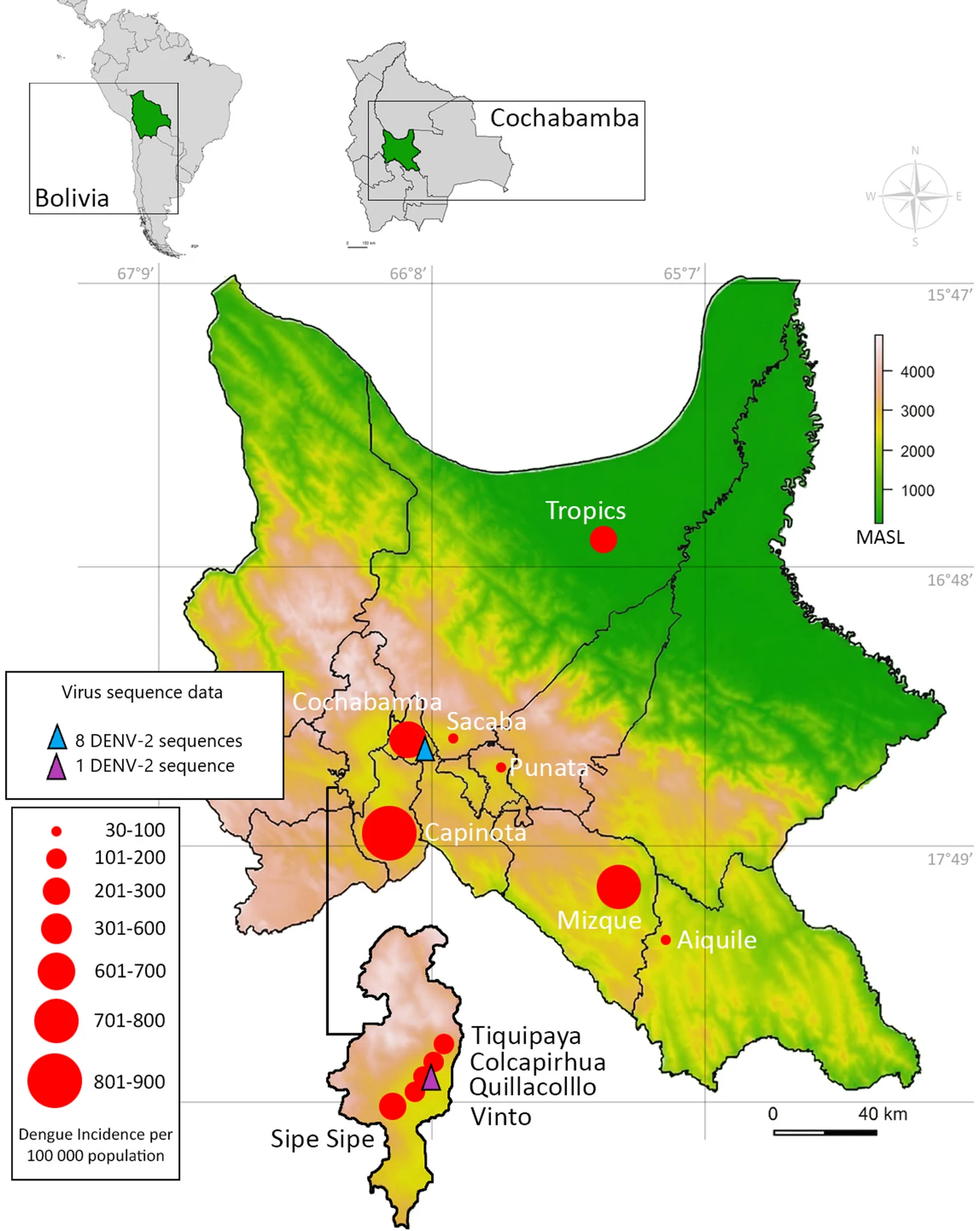
Map of Cochabamba, Bolivia, divided by provinces, showing cumulative estimated dengue incidence per 100,000 population from January to July 2024. Dots represent the estimated incidence in each municipality, and triangles indicate the locations of sequenced samples. The figure was created using Adobe Photoshop with data from GADM maps and data (https://gadm.org/maps/BOL/cochabamba.html)

Bolivia’s geography is divided into three main regions: the Andean highlands, covering 28% of the country with altitudes above 3,000 m; the Sub-Andean valleys and Yungas, comprising 13% of the territory with temperate to warm climates at altitudes of up to approximately 2,500m; and the lowland plains, which account for 59% of the country and feature tropical forests. Administratively, the country is divided into nine departments, including Cochabamba Department, which itself is subdivided into 16 provinces and 47 municipalities [4]. Cochabamba encompasses Sub-Andean valleys and tropical lowlands (Fig. 1) [5]. It is the country’s third most populous department, with approximately 2 million inhabitants. Its capital, Cochabamba city, is situated at 2,558 m and is the department’s most densely populated area, home to 665,505 residents as of 2024 [4].

Cochabamba city, also known as the “Eternal Spring” experiences a semi-arid climate (Köppen: BSk), bordering on a subtropical highland climate (Köppen: Cwb). It is characterized by a dry season from May to October and a wet season from November to March [6]. However, in recent years, it has experienced rising temperatures. Specifically, the spring of 2023, as well as the summer and autumn of 2024, were the hottest on record, with temperatures reaching up to 2 °C above the long-term average [7].

In Bolivia, DENV has been reported circulating since 1931. Seroprevalence studies conducted by 2017 revealed high infection rates in the tropical and subtropical lowlands, including Santa Cruz (93.5%) and Beni (90.0%), and lower rates in highland regions such as Cochabamba (10.4%) and La Paz (11.8%) [8]. In recent years, *Ae. aegypti* has expanded its geographic range to nearly two-thirds of the country, reaching altitudes of up to 2,900 m in the Andes [9–13].

The number of DENV cases also showed a significant increase in Cochabamba, with confirmed cases rising from fewer than 130 before 2018 to 1,400 in 2019. In 2024, the department experienced an unprecedented outbreak, with 13,022 suspected cases and 8,087 confirmed cases reported by December, accounting for 63.2% of Bolivia’s total confirmed infections [3] (Supplementary Fig. 1, Supplementary Fig. 2).

In this study, we collected data on suspected and confirmed DENV cases from the 2024 outbreak in Cochabamba, Bolivia. We performed epidemiological analyses to characterize the spatial and temporal distribution of cases and conducted viral genome sequencing and phylogenetic analyses on a subset of confirmed samples to investigate circulating DENV genetic diversity and their evolutionary relationships.

## Methods

### Data and sample collection

Anonymized data, including age group, sex, department, municipality, month of serum sample collection, and test results, were collected between January 2 and July 15, 2024. Additionally, one milliliter of leftover serum from ten anonymized patients was obtained in June 2024, based on logistical feasibility, by the Departmental Health Service (SEDES) of Cochabamba, the only laboratory in the department authorized to conduct arboviral diagnostics.

### Dengue virus diagnostic testing

Blood samples were collected via venipuncture using serum collection tubes. Following centrifugation, the sera were tested for dengue virus based on the duration of the patient’s symptoms. For individuals presenting within five days of symptom onset, detection was performed using either real-time RT-PCR with Center for Disease Control and Prevention (CDC) primers (DENV-1–4 Real-Time RT-PCR Multiplex Assay [14]) or the commercial nonstructural protein 1 (NS1) antigen enzyme-linked immunosorbent assay (ELISA) test (Euroimmun, Lübeck, Germany). For patients with symptoms lasting more than six days, an in-house MAC-ELISA was used. The collection of samples and the diagnostic tests were conducted at SEDES, Cochabamba.

### Dengue virus sequencing

A specific set of primers (Supplementary Table 1) was used to generate eight overlapping amplicons spanning the entire DENV-2 genome with the Superscript IV One-Step RT-PCR System (ThermoFisher Scientific). PCR mixes (final volume 25µL) contained 3µL of nucleic acid extract, 1.25µL of each primer (10µM), 12.5µL of 2X Platinum SuperFi RT-PCR Master Mix, 6.5µL of RNAse-free water and 0.5µL of SuperScript IV RT Mix. Amplifications were performed under the following conditions: 10 min at 55 °C, 2 min at 98 °C, followed by 40 cycles with the three following steps: 10 s at 98 °C, 10 s at 55 °C and 1.45 min at 68 °C, and a final step at 68 °C for 5 min.

The size of PCR products was verified by gel electrophoresis. For each sample, an equimolar pool of all amplicons was prepared and purified using Monarch PCR & DNA Cleanup Kit (New England Biolabs). After Qubit quantification using the Qubit^®^ dsDNA HS Assay Kit and Qubit 2.0 fluorometer (Thermo Fisher) amplicons were sonicated (Bioruptor^®^, Diagenode, Liège, Belgium) into 250 bp long fragments. Fragmented DNA was used for library preparation using the Ion Plus Fragment Library Kit with the AB Library Builder System (Thermo Fisher). To ensure the equimolar pooling of the barcoded samples, a real-time PCR quantification step was performed using Ion Library TaqMan™ Quantitation Kit (Thermo Fisher). An emulsion PCR of the pools was performed, followed by loading on 530 chips using the automated Ion Chef instrument (Thermo Fisher), and sequencing using the S5 Ion Torrent technology (Thermo Fisher), following manufacturer’s instructions. All sequencing procedures were carried out at the Emerging Viruses Laboratory (UVE) in Marseille, France.

### Viral sequence data analysis

Read data were analyzed with an in-house Snakemake pipeline [15]. Read alignment was achieved using BWA MEM (v0.7.17 [16]) using, as a reference, the best match identified by BLASTing (magicblast, v1.7.7 [17]) sequencing reads using a database of flavivirus sequences including 42 sequences representative of DENV-2 genetic diversity. Consensus sequences were called using the iVar (v1.3.1 [18]) consensus command, and a minimum coverage depth of 50x. Regions with insufficient coverage were masked with N characters. To limit the risk of incorrect indels in homopolymer regions, consensus sequences were curated manually after consensus calling with iVar.

DENV-2 genomes from this study were aligned with a set of reference sequences representative of DENV-2 genotypes. Phylogenetic relationships were inferred using IQ-Tree (version 1.6.12 [19, 20]), with the best-fit model identified by ModelFinder and branch support assessed using an ultrafast approximation (UFBoot2) (1000 replicates). The set of reference genomes used for genotyping was selected based on the set of genomes used in the widely-used tool genome detective to DENV genotyping [21]. For the set of reference genomes to better reflect the phylogenetic diversity within DENV-2 serotype we added the sequence of the highly divergent strain QML22 isolated from a traveler returning from Borneo to Australia [22]. Virus clade was determined using the online Nextclade tool [23] (https://clades.nextstrain.org).

All publicly available sequences for DENV-2 genotype II with a length above 9,500 nt were downloaded from the GISAID database on February, 23rd, 2025 (doi: https://doi.org/10.55876/gis8.250223hf) (Supplementary Text). The sequences were aligned using MAFFT (version 7.51142), trimmed to their coding regions (ORF) and inspected manually.

We then removed potential recombinant sequences from the dataset using the Recombination Detection Program (RDP) version 4. We used RDP, GENCONV and MAXCHI methods for primary screening and BOOTSCAN and SISCAN methods to check for recombination signals [24–28]. We used the automask option to ensure optimal recombination detection.

Using the resulting recombinant-free alignments, we performed ML phylogenetic reconstruction with IQ-Tree (version 1.6.1250), using a GTR + F+R4 model (General time reversible model with empirical base frequencies and a FreeRate model with 5 categories) and assessed branch support using an ultrafast bootstrap approximation (UFBoot2) (1000 replicates).

Finally, we selected a subset of 92 sequences, corresponding to the closest phylogenetic relatives of sequences from Cochabamba 2024, to perform Bayesian inference. We performed a first root-to-tip regression and identified ten outliers that were removed (Supplementary Figs. 4 A and 4B), yielding a final dataset of 82 sequences. Using this dataset, we evaluated the timeframe of the introductions into Cochabamba corresponding to clade A and B (see results), we reconstructed time-scaled phylogenies with BEAST (v1.10.5 [29]), under two different substitution models (the HKY or GTR substitution models with gamma-distributed rate variation among sites and no partition into codon positions), an uncorrelated relaxed clock model, and two different coalescent models (exponential and Bayesian skygrid). We ran single MCMC chains of 100 million states with the BEAGLE computational library [30]. We used Tracer (v1.7 [31]), for inspecting the convergence and mixing, discarding the first 10% of steps as burn-in, and ensuring that estimated sampling size (ESS) values associated with estimated parameters were all > 200. To identify the best-fit model we performed marginal likelihood estimation using path sampling/stepping-stone sampling. All estimates are provided in Supplementary Table 2. Final estimates are provided based on the best model identified. All xml files for these analyses are available at https://github.com/rklitting/D2_Bolivia_2024.

### Statistical Methods

Statistical analyses were conducted using IBM SPSS Statistics version 24.0. Associations between DENV positivity and demographic variables were assessed, and p-values < 0.05 were considered statistically significant. Incidences per 100,000 population and the corresponding 95% confidence intervals (95% CI) were calculated for overall infections and for each municipality using binomial proportion methods.

## Results

### Epidemiological profile of the dengue cases

A total of 9,576 suspected DENV cases were tested at SEDES, Cochabamba. Of these, 5,923 were positive by at least one diagnostic method, resulting in an overall positivity rate of 61.9% (Table 1) and an estimated incidence of 294 (95% CI: 286–301) per 100,000 population. Among the confirmed cases, females accounted for 56.0% (3,314/5,920; *p* < 0.01).

**Table 1:** Characteristics of patients with suspected and confirmed dengue infection in Cochabamba, Bolivia

Result*	No. Population (census 2024[4])	No. Positive / No. Total suspected cases (%)	No. Positive / No. Total confirmed cases (CC) (%)
Positive	2,016,357	5923/9576 (61.9)	NA
Negative	2,016,357	3653/9576 (38.1)	NA

Sex	No. Population (census 2024[4])	No. Positive / No. Total suspected cases (%)	No. Positive / No. Total CC (%)
Female	1,023,071	3314/9568 (34.6)	3314/5919 (56.0)
Male	993,286	2605/9568 (27.2)	2605/5919 (44.0)

Municipality/Province (meters above sea level)	No. Positive / (census 2024[4])	No. Positive / No. Suspected cases per city (%)	No. Positive / No. Total CC (%); Incidence x 100,000 (95% CI)
Cochabamba (2,558 m)	665,505	4104/6404 (64.1)	4104/5923 (69.3); 617 (598–635)
Quillacollo (2,422 m)	165,830	300/531 (56.5)	300/5923 (5.1); 181 (160–201)
Capinota (2,386 m)	31,347	263/390 (67.4)	263/5923 (4.5); 839 (738–940)
Sacaba (2,719 m)	218,502	181/328 (55.2)	179/5923 (3.1); 83 (71–95)
Mizque (2,007 m)	19,846	151/233 (64.8)	151/5923 (2.5); 761 (640–882)
Sipe Sipe (2,550 m)	55,601	117/195 (60.0)	117/5923 (2.0); 210 (172–249)
Tiquipaya (2,649 m)	61,600	72/135 (53.3)	72/5923 (1.2); 117 (90–144)
Vinto (2,553 m)	55,773	71/134 (53.0)	71/5923 (1.2); 127 (98–157)
Colcapirhua (2,561 m)	66,235	70/123 (56.0)	70/5923 (1.2); 106 (81–130)
Punata (2,552 m)	34,813	14/27 (51.8)	14/5923 (0.2); 40 (19–61)
Aiquile (2,250 m)	23,000	8/20 (40.0)	8/5923 (0.1); 35 (11–59)
Tropics: Villa Tunari/Chimoré/Shinahota/Puerto Villarroel/ Entre Ríos (~300 m)	259,894	524/745 (70.3)	524/5923 (8.8); 202 (184–219)
Others	NA	49/300 (16.3)	49/5923 (0.8); NA

Month (year 2024)		No. Positive / No. Suspected cases per month (%)	No. Positive / No. Total CC (%)
January		241/392 (61.5)	241/5923 (4.1)
February		334/562 (59.4)	334/5923 (5.6)
March		691/1182 (58.5)	691/5923 (11.7)
April		1803/2880 (62.6)	1803/5923 (30.4)
May		2243/3456 (64.9)	2243/5923 (37.9)
June		575/994 (57.8)	575/5923 (9.7)
July		36/110 (32.7)	36/5923 (0.6)

Age-range (years)	No. Positive / (census 2024[4])	No. Positive/ No. Suspected cases per age (%)	No. Positive / No. Total CC (%)
0-14	533,848	1032/1910 (54.0)	1032/5902 (17.5)
15-64	1,320,028	4420/6883 (64.2)	4420/5902 (74.9)
>65	162,481	450/742 (60.6)	450/5902 (7.6)

*Nonstructural protein 1 (NS1) antigen enzyme-linked immunosorbent assay (ELISA), in house MAC-ELISA, and/or real-time reverse transcription polymerase chain reaction (RT-PCR) tests

Abbreviations: NA, not applicable; m: meter above sea level; others: Arani, Arque, Anzaldo, Arbieto, Ayopaya, Cliza, Cocapata, Colomi, Omereque, Pojo, San Benito, other departments

The denominator in each cell represents the total number of individuals with available data for that category. Missing or unrecorded data were excluded from the calculations

By municipality, among high-altitude cities, Cochabamba (2,558 m) reported the highest number of confirmed cases (4,104/5,923; 69.3%), corresponding to an estimated cumulative incidence of 617 cases per 100,000 population (95% CI: 598–635), followed by Quillacollo (2,422 m), which accounted for 5% of cases (300/5923) and an incidence of 181 per 100,000 population (95% CI: 160.4–201.2) (Fig. 1). However, the highest incidence rates were observed in Capinota (2,386 m; 839 cases per 100,000 population, 95% CI: 738.4–940.2) and Mizque (2,007 m; 761 per 100,000 population, 95% CI: 640.0–882.0) (Fig. 1).

In the tropical lowland regions, including Villa Tunari, Chimoré, Shinahota, Puerto Villarroel, and Entre Ríos, a total of 524 cases were reported, representing 8.8% of the total cases.

Temporal analysis revealed peaks in April and May, which accounted for 30.4% and 37.9% of confirmed cases, respectively.

Age-stratified analysis showed that adults aged 15–65 years represented the largest proportion of confirmed cases (74.9%).

### Outbreak molecular characterization

A total of 437 positive samples were previously serotyped at SEDES, of which five were identified as DENV-1 and 432 as DENV-2.

To determine the genotype(s) of the virus lineages circulating in Cochabamba in 2024, ten positive samples were randomly selected and serotyped, all identified as DENV-2. Sequencing of these samples yielded nine nearly complete DENV-2 genomes (> 80% of the coding DNA sequence [CDS], pre-sequencing amplification failed for the last one). Eight were from Cochabamba city (GenBank accession numbers PV426482- PV426487, PX959620 and PX959622) and one was from Quillacollo city (GenBank accession number PV426488) (Fig. 1, detailed in Supplementary Table 3), all collected in June 2024.

The phylogenetic relationships of these DENV-2 genomes from Cochabamba, 2024, were analyzed together with a set of reference sequences representative of DENV-2 genotypes, and the online Nextclade tool [23] was used to confirm the genotypes and determine their major and minor clades. All genomes belonged to genotype II, clade F.1.1.2 (Supplementary Table 3, Supplementary Fig. 3), consistent with previously reported Bolivian sequences from tropical regions in 2023 available in GISAID, and aligning with DENV-2 clades reported recently in Latin America [32–34].

To assess whether the 2024 Cochabamba outbreak was linked to DENV-2 lineages circulating in Bolivia in 2023, a phylogeny was inferred using a dataset that combined all Cochabamba genomes with all DENV-2 genotype II genomes available in GISAID as of February 23, 2025. The initial dataset included 7,409 genomes from 52 countries and territories (Supplementary Text). Near-complete genomes (length > 9,500 nt) were selected and combined with our sequences for phylogenetic inference using a maximum-likelihood approach with IQ-TREE [19].

**Fig. 2:**
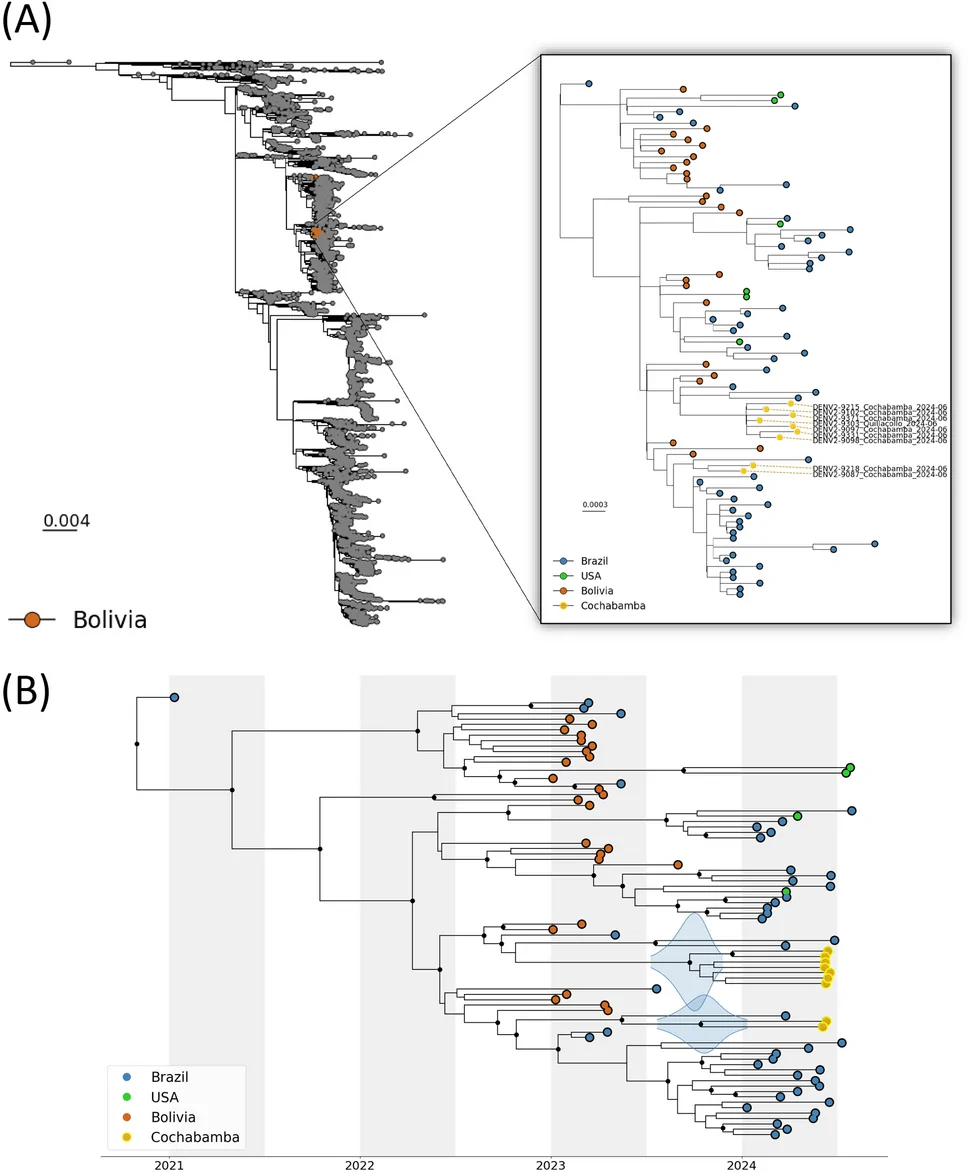
Divergence (**A**), and time-resolved (**B**) phylogenies of DENV2 sequences from the 2024 Cochabamba outbreak. **A**. Maximum-Likelihood phylogeny of all DENV2-II near-complete (> 9,500 nucleotides) genomes available from GISAID February, 23rd, 2025 (doi:https://doi.org/10.55876/gis8.250223hf) combined with the nine DENV2 sequences produced in the study from the Cochabamba outbreak, and a set of reference sequences representative of DENV2 genotypes. Phylogenetic inference was performed using IQTREE2 under a GTR + F+R5 substitution model with ultrafast bootstrap approximation (1000 replicates). The tree was rooted using the sylvatic genotype of DENV2. In the subtree, sequences from the Cochabamba outbreak are highlighted in yellow, those from Bolivia, in orange, from Brazil in blue and those from the USA in green. Bootstrap values are shown for all nodes with value above 95. **B.** Maximum clade credibility (MCC) tree of DENV genomes from the Cochabamba outbreak and their closest phylogenetic ancestors. The blue violin plots indicates the 95% highest posterior density (HPD) interval for the tMRCA of clade A and B. Nodes with posterior support above 0.9 are shown with a black dot

The resulting phylogenetic tree (Fig. 2A) showed that the 2024 Cochabamba sequences formed two distinct clades (A and B) with strong statistical support (bootstrap > 95). Although both clades A and B were phylogenetically close to sequences sampled in 2023 from Santa Cruz, Bolivia (tropics), clade A was rooted by a sequence from the state of Paraná, Brazil, sampled in 2024 (Curitiba, hDenV2/Brazil/SP-IAL-357154993/2023), whereas clade B was rooted by two other sequences from Brazil sampled in 2024, in the states of Paraná (hDenV2/Brazil/PR-Fiocruz-LRV24H3138/2024, Cascavel) and Santa Catarina (hDenV2/Brazil/SC-LACENSC-422083052/2024, São Francisco do Sul).

To provide a timeframe for the emergence of clades A and B in the department of Cochabamba, Bayesian inference was performed on a subset of 82 sequences corresponding to the nine sequences generated in this study combined with their closest phylogenetic relatives (Fig. 2B). We estimated that clade A probably emerged no later than late 2023 (95% Highest Posterior Density interval (HPD): [2023-07;2023-11], and clade B no later than January 2024 (95% HPD: [2023-07;2024-01]).

## Discussion

In 2024, the Sub-Andean valleys of Cochabamba’s Department, Bolivia, at ~ 2,550 m above sea level, experienced their first large-scale dengue virus outbreak [3].

In this study, we analyzed data from 5,923 laboratory-confirmed infections between January and July 2024, representing 73.2% of all cases reported in the department that year, and performed sequencing and phylogenetic analysis on a set of nine samples. To our knowledge, this is the first study to characterize the epidemiological and molecular features of the 2024 dengue outbreak in Cochabamba.

Our analysis highlights substantial virus circulation across municipalities at altitudes up to 2,719 m, from densely populated cities such as Cochabamba city (665,505 inhabitants) to smaller towns such as Aiquile (23,000 inhabitants). Most cases occurred in Cochabamba city (4,104), while the highest incidence was observed in Capinota (839 cases per 100,000 population).

The temporal distribution of cases peaking in April and May, corresponds to the end of the rainy season, reinforcing the well-established link between precipitation, vector density and dengue transmission dynamics [35]. The economically active age groups were more affected, likely due to behavioral and occupational factors that increase exposure to mosquito bites in outdoor or communal settings such as workplaces [36]. The predominance of female cases (56%) may reflect gender-related differences in exposure patterns or healthcare-seeking behavior, with women potentially more likely to seek medical attention [37].

The presence of *Ae. aegypti* at high altitudes in Bolivia has been continuously documented since 2016, reaching elevations of up to 2,900 m [9–13], and the record-high temperatures observed at the end of 2023 and in 2024, reaching up to 38.3 °C [38], may have enhanced mosquito survival, accelerated development and reproduction, increased population abundance, and shortened the extrinsic incubation period of the virus. In addition, other environmental and anthropogenic factors, such as urbanization, deforestation, and increased human mobility between endemic regions within the country and from neighboring countries [33, 39], may also have collectively influenced DENV transmission dynamics and likely promoted the outbreak observed that year.

Molecular analyses identified the circulation of two DENV serotypes (DENV-1 and DENV-2), with a predominance of DENV-2 (Cosmopolitan), consistent with patterns reported in several other countries in the region [40, 41]. Phylogenetic inference revealed the presence of two distinct clades within Cochabamba, indicating that multiple, closely related DENV-2-II F.1.1.2 might have contributed to the outbreak.

Bayesian inference estimates indicate that the DENV-2 clades identified during the Cochabamba outbreak emerged at the latest in late 2023 early 2024, aligning with the timing of increase in cases that marked the beginning of the outbreak. These results suggest that DENV-2 transmission in 2024 in Cochabamba stemmed from recent introductions into the department with limited cryptic circulation prior to detection.

Cochabamba DENV sequences were rooted with recent virus strains from Brazil, specifically from the southern region, which generally has a more temperate climate compared to the tropical north. The sequences originated from two cities in the state of Paraná: Curitiba, at 930 m (Cfb Köppen climate), characterized by cooler summers, and mild winters, and Cascavel at 780 m (Cfa Köppen climate) [42], with a humid subtropical climate and cooler winters; as well as São Francisco do Sul in the State of Santa Catarina, which has humid coastal subtropical/tropical climate [43]. The circulation of DENV-2-II F.1.1.2 in these areas raises questions regarding its evolutionary trajectory and suggests that this lineage may have acquired features enhancing its fitness and capacity for local transmission. Future studies, including comprehensive genomic analyses and assessment of vector competence, could provide further insights into the mechanisms underlying its emergence and adaptation.

However, due to the limited availability of recent DENV-2 genomic data from Bolivia and neighboring countries, including Paraguay and Argentina, the precise origin and routes of introduction of these viral lineages into the department of Cochabamba cannot be confidently determined.

This study has several limitations that should be considered when interpreting the findings. First, the relatively small number of sequenced samples likely does not represent the full genetic diversity of DENV circulating during the outbreak, and the fact that samples were collected after the epidemiological peak and primarily from Cochabamba city introduces both temporal and spatial sampling biases. This clustering limits our capacity to accurately infer viral evolutionary dynamics, estimate the timing and frequency of viral introductions, and reconstruct patterns of geographic spread across the department and, potentially, at the national level. Second, the reliance on passive surveillance data may underestimate the true burden of disease, as dengue infections are frequently underdiagnosed and underreported, particularly in cases with mild or atypical clinical presentations. Additionally, uneven sampling across municipalities may have further restricted the detection of transmission hotspots.

Future work should integrate vector surveillance with viral sequencing, seroprevalence studies, and climatic data to build a robust high-altitude dengue transmission model. Despite these limitations, this study provides comprehensive insights into the geographic expansion of DENV transmission in Bolivia and addresses a critical data gap in Cochabamba, representing the first study to characterize dengue dynamics in this setting.

A multifaceted approach is essential, including sustainable vector control strategies, public health education, and coordinated monitoring of viral diversity at regional and international levels. Additionally, addressing climate change must be considered a critical component of dengue prevention, as measures such as promoting urban green infrastructure, reducing greenhouse gas emissions, and limiting deforestation can help lower long-term dengue transmission risk and strengthen overall public health resilience [39].

## Conclusion

The 2024 dengue outbreak in Cochabamba represents a significant epidemiological event, illustrating that dengue is increasingly emerging at high-altitudes and underscoring the growing challenges of disease control. Our findings reveal substantial local transmission, multiple virus introductions, and the vulnerability of high-altitude populations to emerging arboviral threats. This study provides essential data to guide public health interventions and preparedness for future arboviral outbreaks in Cochabamba and similar high-altitude settings.

## Supplementary Information


Supplementary Material 1


## Data Availability

The data supporting the findings of the study are available within the manuscript and the supplementary information. Genome sequences generated in this study have been deposited in GenBank under accession numbers PV426482-PV426488; PX959620 and PX959622.
