# Supplementary material for: Emergence of dengue at high altitude: characterization of the 2024 outbreak in Cochabamba, Bolivia
Source: Virol J. 2026 Mar 3;23:90. doi: 10.1186/s12985-026-03117-1 (PMC13045065; doi:10.1186/s12985-026-03117-1)
Supplement: Supplementary file 1 — Supplementary Material 1 [file 12985_2026_3117_MOESM1_ESM.pdf]

Emergence of dengue at high altitude: Characterization of the 2024 outbreak in Cochabamba, Bolivia

Supplementary Material

Supplementary Text. Data availability and data snapshot

GISAID Identifier: EPI_SET_250223hf; doi: 10.55876/gis8.250223hf

All genome sequences and associated metadata in this dataset are published in GISAID's EpiArbo database. To view the contributors of each individual sequence with details such as accession number, Virus name, Collection date, Originating Lab and Submitting Lab and the list of Authors, visit 10.55876/gis8.250223hf

EPI_SET_250223hf is composed of 7,409 individual genome sequences.

The collection dates range from 1982-01-01 to 2025-01-20;

Data were collected in 52 countries and territories;

All sequences in this dataset are compared relative to the official reference sequence employed by GISAID.

Supplementary Table 1. Amplification primers used for whole genome sequencing of DENV2.

Forward primers	Sequence (5' to 3')	Reverse primers	Sequence (5' to 3')
D2_F1	agtwgttagtctacgtgsaccgac	D2_antilles_R1_rc	catwgcacaggtcacratrcc
D2_antilles_F2	ctccatggtagayagaggatg	D2_R2	caacacaaayagagcttggaaytc
D2_F3	atyatgcaggcaggaaaacgatc	D2_antilles_R3_rc	gtgcagctcacyttccatgc
D2_antillesF4	gcagctggactactcttaag	D2_antilles_R4_rc	gagttgagatgtatcctctagcygctat
D2_antilles_F5	gaggacttccrataagataycaaacc	D2_R5_rc	cagtattattgaagctgctatcc
D2_antilles_F6	cgtacaatcaygctcttagtg	D2_R6_rc	gtgaygaytcccctatgtcacac
D2_F7	cctaacaaaaggaggaccaggac	D2_antilles_R7_rc	tctcgatatratgtccattactg
D2_antilles_F8	gtgcaaagaccgacaccaag	D2_R8_rc	agaacctgttgattcaacagcacc

Supplementary Table 2. TMRCA estimates from BEAST analyses under different substitution models and coalescent tree priors. Shown for each coalescent tree prior is the median, with the 95% highest probability distribution of TMRCA in parentheses. Also shown is the log marginal likelihood obtained using path-sampling and stepping-stone sampling for each model/prior combination.

Substitution model	Tree prior	Median clade A	95%HPD – clade A	Median clade B	95%HPD – clade B	Log marginal likelihood (PS)	Log marginal likelihood (SS)
HKY+G4	Exp. growth	2023-09-17	[2023-05-24;2023-12-22]	2023-08-21	[2023-03-24;2024-01-06]	-19812.3268	-19812.3756
GTR+G4	Exp. growth	2023-09-15	[2023-05-23;2023-12-24]	2023-08-22	[2023-03-26;2024-01-09]	-19740.6956	-19740.5242
HKY+G4	Skygrid	2023-09-29	[2023-07-11;2023-11-25]	2023-10-19	[2023-07-22;2024-01-12]	-19786.3754	-19787.0721
GTR+G4	Skygrid	2023-09-28	[2023-07-10;2023-11-24]	2023-10-20	[2023-07-22;2024-01-11]	-19728.1511	-19729.4009

Supplementary Table 3. Sequence data and corresponding metadata for the ten sequenced samples from Cochabamba, Bolivia. For each sample, the percentage of the genome covered is given for a depth of 50X. Virus clade was determined using the online Nextclade tool (https://clades.nextstrain.org).

Sample number	Virus	Sampling year-month	Country of origin	Geographical details Department (Province)	GenBank Accession number	% genome covered	Genotype	Clade
9303	DENV2	2024-06	Bolivia	Cochabamba (Quillacollo)	PV426488	99.38	II (Cosmopolitan)	F.1.1.2
9102	DENV2	2024-06	Bolivia	Cochabamba	PV426487	99.43	II (Cosmopolitan)	F.1.1.2
9098	DENV2	2024-06	Bolivia	Cochabamba	PV426486	99.4	II (Cosmopolitan)	F.1.1.2
9097	DENV2	2024-06	Bolivia	Cochabamba	PV426485	99.43	II (Cosmopolitan)	F.1.1.2
9087	DENV2	2024-06	Bolivia	Cochabamba	PV426484	99.43	II (Cosmopolitan)	F.1.1.2
9215	DENV2	2024-06	Bolivia	Cochabamba	PV426482	84.87	II (Cosmopolitan)	F.1.1.2
9218	DENV2	2024-06	Bolivia	Cochabamba	PV426483	90.57	II (Cosmopolitan)	F.1.1.2
9331	DENV2	2024-06	Bolivia	Cochabamba	PX959622	84.19	II (Cosmopolitan)	F.1.1.2
9371	DENV2	2024-06	Bolivia	Cochabamba	PX959620	98.3	II (Cosmopolitan)	F.1.1.2

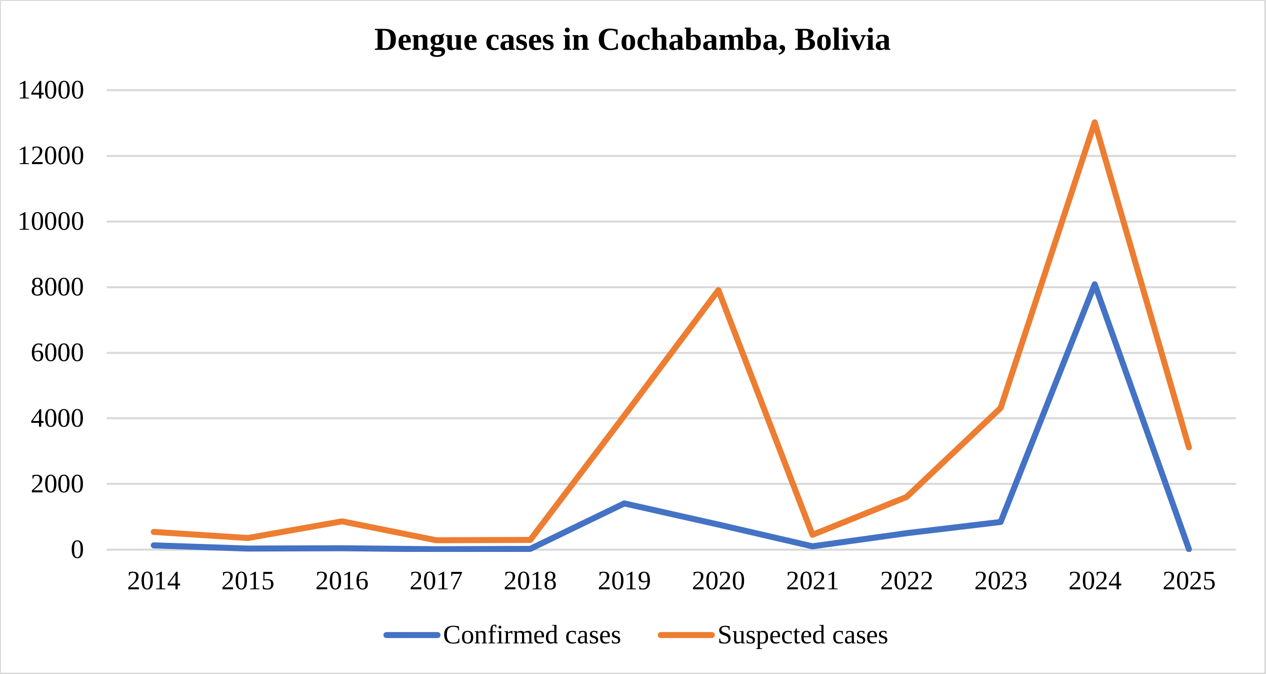

Supplementary Figure 1: Confirmed and suspected dengue virus cases in the Department of Cochabamba, 2014–2025 (PAHO)

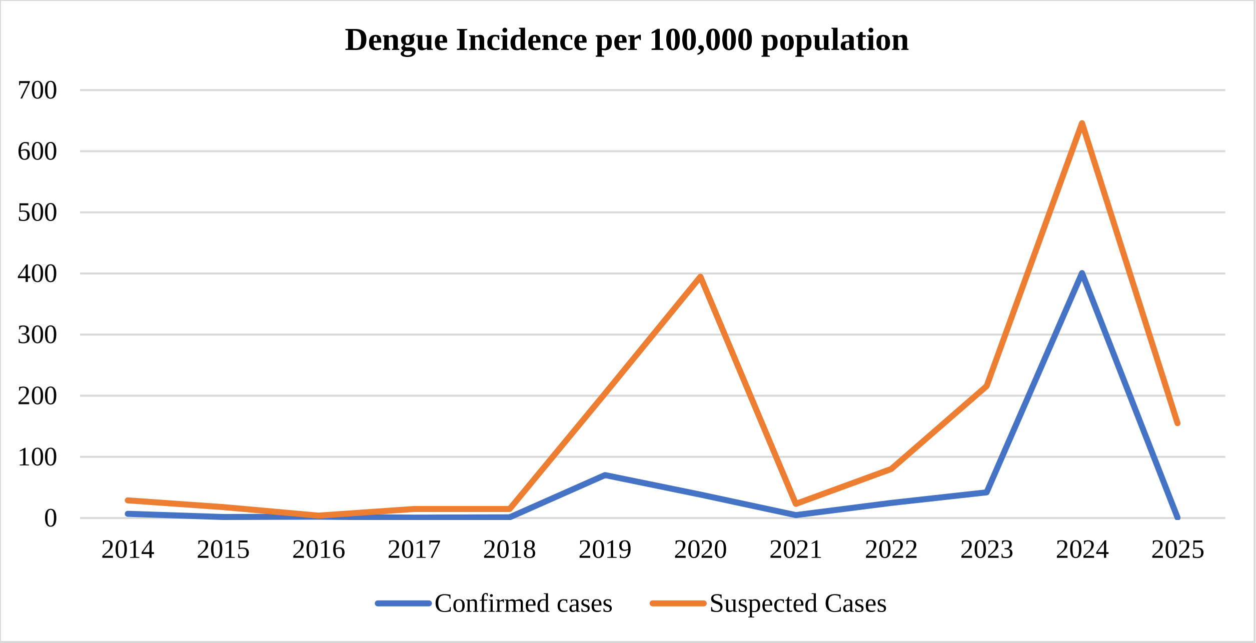

Supplementary Figure 2: Dengue virus incidence per 100,000 population in the Department of Cochabamba, 2014-2025 (PAHO).

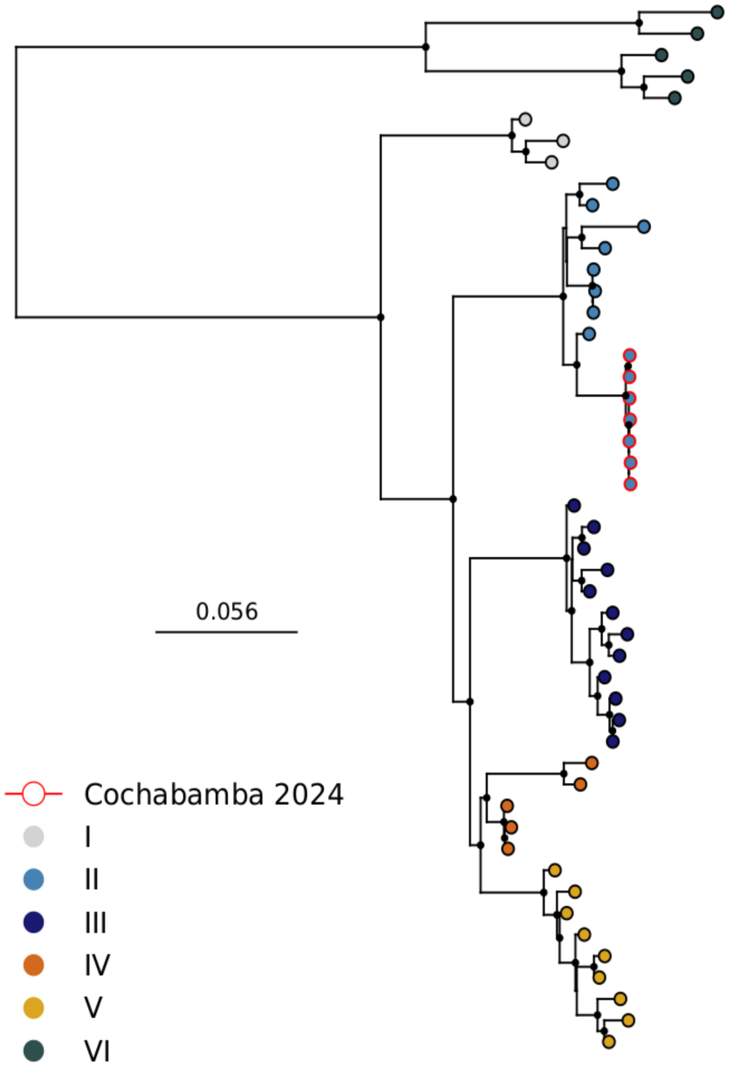

Supplementary Figure 3. Maximum-Likelihood phylogeny of all DENV-2 sequences from the Cochabamba 2024 epidemic with a set of reference sequences representative of DENV-2 genotypes Phylogenetic inference was performed using IQTREE2 under the best substitution model identified by ModelFinder with ultrafast bootstrap approximation (1000 replicates). The tree was rooted using the highly divergent strain QML22 (KX274130). Nodes with bootstrap support above 95 are highlighted with a black circle.

A)

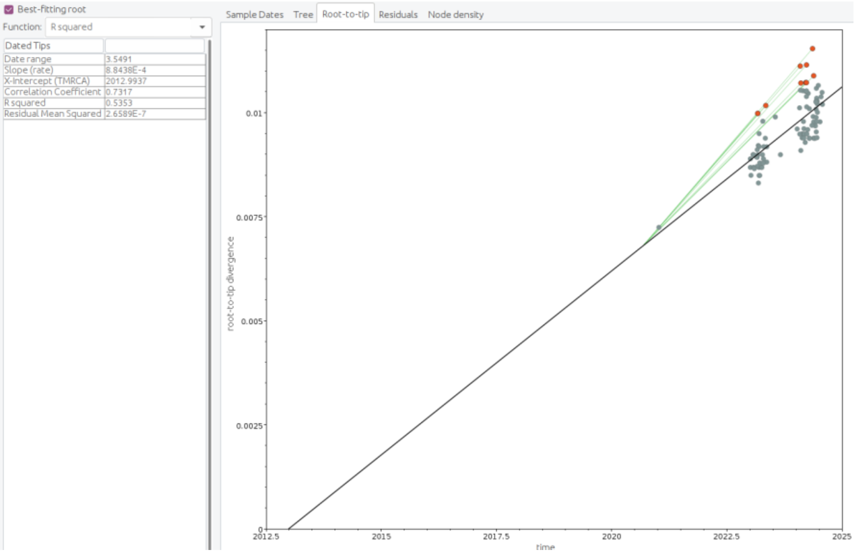

B)

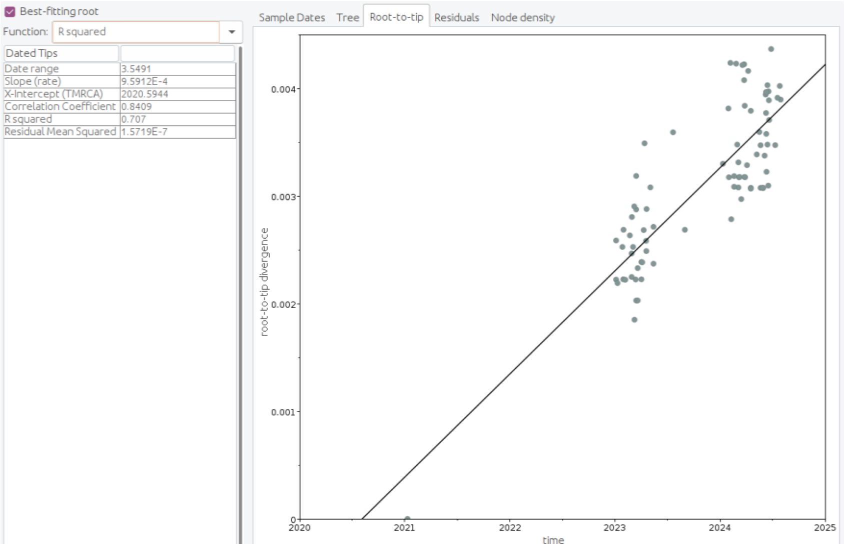

Supplementary Figure 4. Root-to-tip regression on subsets of 92 (A) and 82 (B) sequences selected for Bayesian inference. Regression of genetic distance against time for all sequences included in the dataset used for Bayesian inference. The outliers excluded from the final analysis in panel A are shown as red circles.
